# Multiplexed Analysis of Multicomponent Biomolecular Condensates without Any Tag

**DOI:** 10.1021/jacs.5c14476

**Published:** 2025-12-08

**Authors:** Gyula Pálfy, Johannes Schmoll, Maria E. Pérez, Fred F. Damberger, Yaning Han, Leonidas Emmanouilidis, Frédéric H.-T. Allain, Mihajlo Novakovic

**Affiliations:** Department of Biology, Institute of Biochemistry, 27219 ETH Zurich, 8093 Zurich, Switzerland

## Abstract

Biomolecular condensates, formed in the process of liquid–liquid phase separation (LLPS), play key roles in RNA metabolism and cellular organization. These dynamic assemblies often contain several components, such as proteins and RNAs. Experimental methods to study biological condensates commonly involve fluorophore-labeling of various droplet components, which may affect phase separation behavior and are thus not able to probe biomolecules in their chemically native state. Here, we introduce a noninvasive, multiplexed NMR approach that enables selective observation of multiple components in hydrogel-stabilized biphasic condensates without using tags. The introduced multiplexing filter combines enhanced spin–spin cross-relaxation in the condensed phase with diffusion and isotope filters to resolve signals from distinct protein pools from both the dilute and condensed phases. We demonstrate the robust performance of this 1D ^1^H NMR experiment using biphasic samples containing condensates of FUS N-terminal domain, FUS full-length, and the multicomponent condensate formed by two intrinsically disordered regions of hnRNPC1 protein with highly overlapping resonances. Integrating the multiplexing filter into multidimensional experiments enables site-specific structural and dynamic insights from diverse protein populations, extending NMR applications in LLPS.

## Graphical abstract

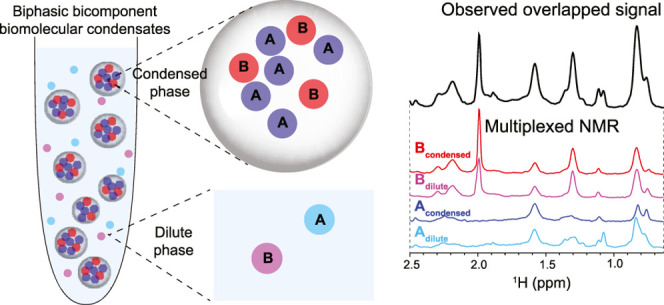

## Introduction

Cells rely on the formation of biomolecular condensates to organize and regulate a crowded cellular milieu.^1,2^ Involved in RNA metabolism, biomolecular condensates are often enriched in proteins that contain RNA-binding domains and LLPS-prone intrinsically disordered regions (IDRs).^3–6^ Fluorescence microscopy has emerged as a powerful method of choice to study condensate morphology and dynamics in both cells and in vitro. However, recent reports suggest that fluorescent tags can affect the LLPS behavior of biomolecules and thus bias interpretation,^7–10^ especially in multicomponent systems where multiple spectroscopically distinct tags need to be used simultaneously for multicolor analysis of individual components.

Solution NMR spectroscopy offers a tag-free methodology to probe structure, dynamics, and interactions in complex environments. However, most NMR studies of biological condensates have focused solely on the macroscopic condensed phase, obtained by centrifugation of biphasic mixtures.^11–14^ Although providing important structural insights, this lacks the droplet morphology observed in vivo and does not capture the dynamic equilibrium between coexisting phases at their interface.^15,16^ As this interface can play a crucial role in condensate aging,^17–19^ associated with disease progression, it is important to study biomolecular condensates noninvasively in their native biphasic environment. Cytoskeleton-mimicking agarose hydrogels can effectively stabilize liquid droplets,^16,20,21^ limiting their size to near-physiological ranges and allowing prolonged spectroscopic analysis without sedimentation. In addition, the preparation of biphasic samples requires much smaller protein quantities compared to macroscopic condensed phase samples. However, in biphasic samples, the spectral overlap of ^1^H signals stemming from the two phases and different protein constituents complicates NMR analysis. Here, we introduce a universal pulse sequence block with multiplexing capabilities that can select different components of biomolecular condensates and can be combined with most of the multidimensional NMR experiments used to probe the structure and dynamics. Utilizing the very different cross-relaxation rates of the two phases, their diffusion contrast, and isotope labeling, our multiplexing filter can resolve the spectra of at least four different molecular states in a biphasic multicomponent system, providing more complete insights into multicomponent condensates.

## Experimental Section

### Sample Preparation

FUS NTD low-complexity domain was expressed and purified as previously described.^20^ The protein sample was prepared at a concentration of 200 μM for NMR analysis in 30 mM HEPES buffer with 200 mM KCl at pH 7.5 with a residual urea concentration of 0.6 M. The phase separation was induced by diluting 10-fold the 2 mM protein stock that was solubilized by 6 M urea. FUS FL protein was also expressed as previously described.^16^ Similarly, 1.5 mM protein stock was diluted 10-fold to a final concentration of 150 μM for NMR analysis in 5 mM phosphate and 5 mM HEPES at pH 7, 1 mM TCEP, 0.6 M urea, and 15 mM NaCl. During the dilution step, agarose containing buffer at 55 °C was added to yield the final agarose mass concentration of 0.5%. Agarose gelation occurs shortly after transferring to the NMR tube.^20^

IDR1 and IDR2 of human hnRNPC1 (Uniprot accession number P07910) were expressed in *Escherichia coli* BL21 Star (DE3) or Rosetta (DE3) bacteria strains after transformation with the plasmids prepared by cloning the respective regions into pTEM vectors using His_6_-GB1 tag (IDR1: 89–179 residues, IDR2: 207–293 residues of hnRNPC1). The cells were grown at 37 °C in M9 medium containing either ^15^N-NH_4_Cl and ^12^C-glucose (IDR1) or ^14^N-NH_4_Cl and ^13^C-glucose (IDR2) until OD_600_ reached 0.6, then expression was induced by 0.1 mM IPTG for 5–6 h at 30 °C. Proteins were then purified by Ni^2+^-affinity chromatography in 50 mM TRIS and 1 M NaCl (pH 8) buffer and eluted with gradually increased imidazole in 100–500 mM range; afterward His-tag was removed by TEV protease overnight, while it was dialyzed against buffer containing 50 mM HEPES pH 6.5, 150 mM NaCl. The completely processed sample was applied to the Ni^2+^-affinity column again, and the flow-through was collected and concentrated to 3–4 mM. The NMR sample contained 400 μM ^15^N-labeled IDR1 and 200 μM ^13^C-labeled IDR2 in 50 mM NaCl in 50 mM HEPES, pH 6.0. The samples containing different protein ratios and high salt concentrations were prepared as specified in Figure S4. During the mixing of the two proteins, low-melting agarose containing buffer was added at 42 °C, resulting in a final concentration of 0.5%. Agarose gelation occurs shortly after transferring to the NMR tube.^20^ All NMR samples contained 5% D_2_O.

### NMR Spectroscopy

All NMR experiments were recorded with a 3 mm NMR sample tube at 298 or 288 K on 700 MHz Avance NEO spectrometers equipped with TCI cryo-probes with *xyz*-gradient system, 900 MHz Avance 3 HD equipped with a TCI cryo-probe, and 1.2 GHz Avance NEO equipped with a 3 mm TCI cryo probe. Experiments were run on TopSpin 3.6, 4.2, or 4.5, and all data were processed using TopSpin 4.5 software (Bruker).

The magnetization transfer (MT) filter was performed by using cw saturation with a duration of 0.5–5 s. Given the efficiency of cross-relaxation in the condensed phase, 0.5–1 s suffices in most cases and should be used as a rule of thumb. When aliphatic protons were observed, the cw irradiation offset was placed at around 8.5 ppm, and conversely, for amide detection, the offset was between 0 and 1.5 ppm. Irradiation at 0 ppm showed a better performance. To ensure the proper bandwidth of saturation, the cw nutation field was varied from 500 to 1000 Hz. Although ca. 800 Hz was sufficient, we used 1000 Hz for the sake of consistency. To switch off the MT filter, far off-resonance saturation at −100,000 Hz (−143 ppm at 700 MHz) was used. For the reference scan used in ^15^N–^1^H HSQC difference spectroscopy at 1.2 GHz, cw saturation was applied at around 16 ppm, downfield from amides, to correct for spurious saturation effects arising from the on-resonance saturation of aliphatic protons.

The diffusion filter was performed using conventional stimulated echo with bipolar gradients.^22^ To ensure that the fast-diffusing signal stemming from the dilute phase was defocused, a diffusion gradient strength of 43–45 G/cm was used. In the case of FUS NTD, the diffusion gradient time was 10 ms, with a total diffusion time of 75 ms.^16^ In the case of the multicomponent hnRNPC1 condensate, we used a 12 ms total gradient duration and 50–100 ms of diffusion delay. These numbers should be optimized for each specific sample by using standard DOSY experiments. Smoothed rectangular-shaped gradients SMSQ10.100 were utilized.

A time-shared ^15^N/^13^C double half filter^23^ was implemented, which was combined with excitation sculpting^24^ in the last part of the multiplexing block for water suppression. To calculate delays for scalar coupling evolution, 91 and 140 Hz were used as *J*_NH_ and *J*_CH_ coupling constants, respectively. BUSS decoupling^25^ was utilized on the ^13^C channel, while GARP4 ^26^ was applied on ^15^N.

All 1D experiments were acquired using 32–128 scans and a *d*_1_ recovery delay of 1.5 s. 2D ^15^N–^1^H HSQC correlation experiments were acquired using 32 scans (24 scans on 1.2 GHz), with 200 time increments (384 increments on 1.2 GHz) in the indirect dimension spanning 23 ppm (centered around 118.5 ppm) and a *d*_1_ of 1 s. For the MT filter, 1 s irradiation was used at the specified offsets. Diffusion-filtered ^15^N–^1^H HSQC was acquired using the same parameters as other ^15^N–^1^H HSQC experiments with the difference of using 128 scans to improve the SNR. On the 1.2 GHz, we used 24 scans for the diffusion-filtered ^15^N–^1^H HSQC for direct comparison.

### Simulations

Simulations on the MT and *T*_2_-filter efficiency were performed using home-written MATLAB codes that are available upon suitable request.

## Results and Discussion

Facilitating phase separation, IDRs play a crucial role in biomolecular condensation and are often part of the proteins found in membraneless organelles.^3–5^ IDRs remain disordered in the condensed phase, with side chains being locally highly dynamic, resulting in comparable line widths of their signals stemming from dilute and condensed phases. Additionally, their spectra are often highly overlapped.^12^ Given large differences in diffusion coefficient between the two phases (2–3 orders of magnitude),^12,16,27^ a diffusion filter can be very effective for selecting the signals originating from the slow diffusing molecules in the condensed phase,^20^ but an alternative filter for observing only the dilute phase remains challenging. So-called relaxation filters (i.e., *T*_2_ filter)^28^ to detect solely the component with slower relaxation are not very efficient at suppressing the condensed phase signals. This is because their proton relaxation rates in the condensed phase are often not sufficiently faster compared to those in the dilute phase to provide a strong contrast (Note S1 and Figure S1A). However, the cross-relaxation between the proton spins becomes highly efficient in the condensed phase because of the significantly increased intermolecular contacts and compaction in the dense phase, increasing the effective proton density (Figure 1A).^12,27,29–33^ In addition, the slower global rotational correlation time^12,34^ due to higher viscosity in the condensed phase also boosts the efficiency of cross-relaxation, the ensuing NOE effect^35^ and spin diffusion. This leads to very fast MT^36,37^ in the condensate, allowing one to suppress one class of spins by irradiation of their spectroscopically distinct, cross-relaxing neighbors while preserving most of the resonances of the dilute phase. For example, saturation of all amide protons is much more efficiently transferred to the aliphatic protons within the molecules in the condensed phase, compared to the dilute phase, reducing their intensity and providing an MT contrast^38,39^ between the phases (Note S1 and Figure 1B).

**Figure 1:**
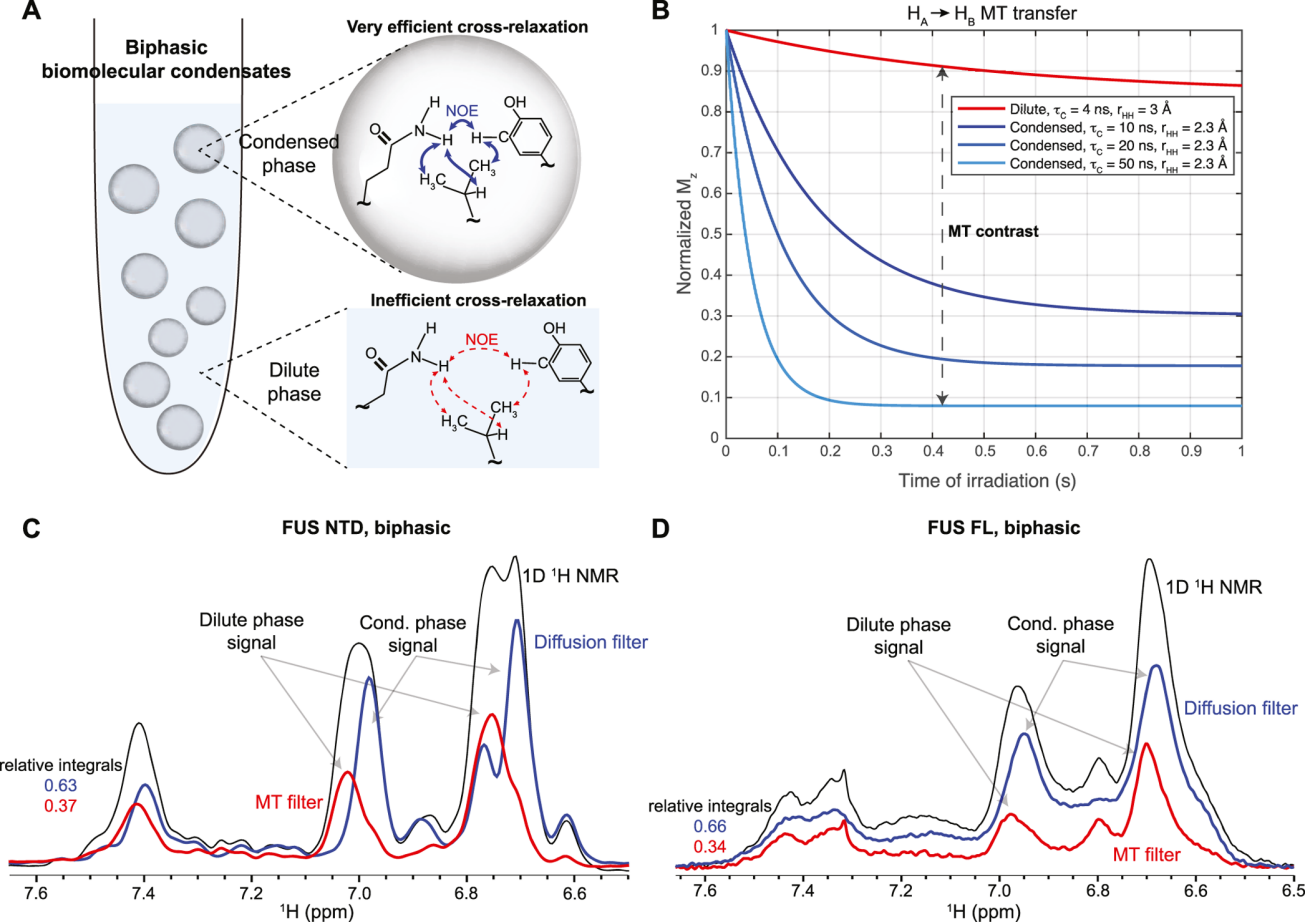
(A) Biphasic biomolecular condensates can be stabilized using a 0.5% (w/v) agarose hydrogel. Due to enhanced intra- and intermolecular contacts, slower molecular tumbling and compaction, cross-relaxation, and the ensuing NOE effect is more efficient in the condensed compared to dilute phase. (B) Simulations performed using the Bloch–McConnell–Solomon equations incorporating a continuous saturation of one proton pool (i.e., amide) and evaluating the ensuing steady-state MT to the other proton pool (i.e., aliphatic) through cross-relaxation. For simplicity, to depict the higher effective proton density, we assumed several combinations of global τ_c_ and average interproton distance *r*_HH_ for medium-sized IDRs in dilute and condensed phase. Increase of τ_c_ and decrease in *r*_HH_ illustrate the effect of enhanced interactions upon condensation. (C,D) The efficiency of the so-called MT filter in selecting the dilute phase signal in FUS NTD and FUS FL sample. Condensed phase acquired using diffusion-filter is shown for completeness. The relative integrals of signals in condensed and dilute phase match well with corresponding protein populations validated using separate experiments. In the MT filter, aliphatic peaks at 0–1 ppm are irradiated with a nutation field of 1000 Hz.

The so-called MT filter has been tested on the biphasic sample of FUS NTD domain, comprising the low complexity QGSY-rich segment and the first arginine–glycine repeat (RGG1), which phase separates at micromolar concentrations and drives phase separation of the FUS protein.^40^ Despite largely overlapping signals in the side chain aromatic region, the MT and diffusion filters can very efficiently resolve the FUS NTD signal stemming from the dilute and condensed phases (Figure 1C). As illustrated in Figure S1B, irradiation of aliphatic protons upfield from water selectively saturated 80% of the condensed phase signal while not notably reducing the dilute phase signal. These experimental data agree with predictions in Figure 1B, suggesting that our simulations are an adequate tool to assess the efficiency of diffusion- and MT-filter experiments for condensed and dilute molecular systems. We further confirmed the robust performance of the MT filter on a FUS full-length (FUS FL) biphasic sample (Figures 1D and S1C). In both cases, the relative peak integrals of FUS NTD and FUS FL in dilute and condensed phases are in line with protein populations as determined by our REDIFINE approach^16^ (Figure S1D) and can therefore be used semiquantitatively as an estimation of relative protein partitioning (Note S1). The efficiency and selectivity of the filter experiments remain excellent in the absence of agarose (Figure S1E,F), although continuous sedimentation of the condensed phase compromised the sample stability over time.

Most biologically relevant condensates contain multiple components. This increases sample complexity, as the coexisting dilute and condensed phases contain different pools of the protein or RNA molecules. To deconvolve different constituents, we combined diffusion and MT filters together with an isotope filter/edit block^23^ within a single pulse sequence (Scheme 1A). Utilizing differentially ^15^N- and ^13^C-labeled compounds, this multiplexing filter can be used in a 1D ^1^H experiment, which allows the selection of the desired component of a biphasic sample by combining the different filters (Scheme 1B). The pulse sequence is described in detail in Note S2 and Figure S2.

**Scheme 1:**
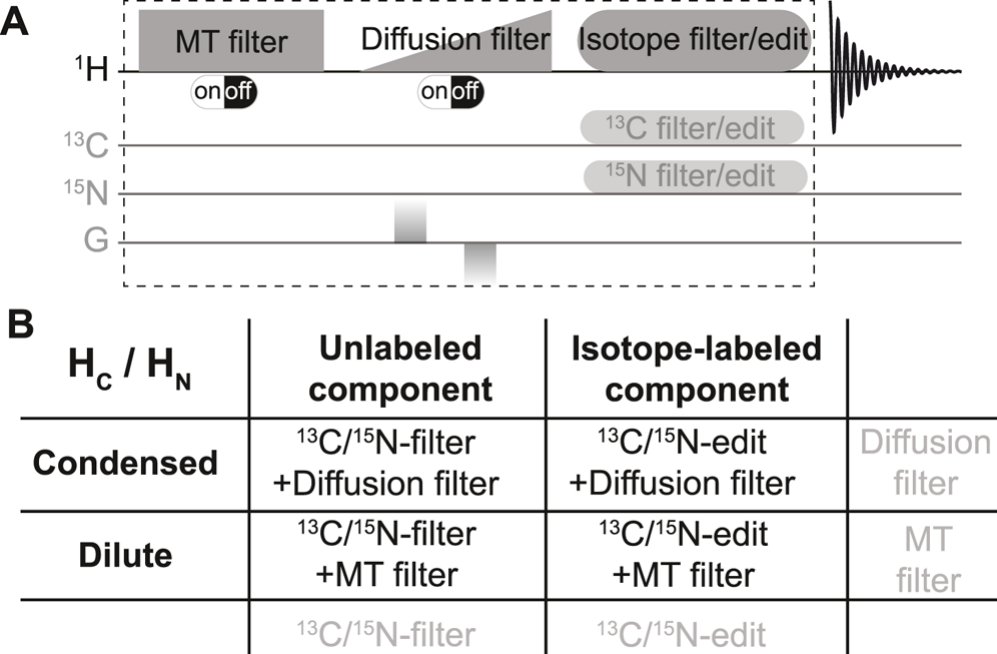
(A) Illustration of the Multiplexing Filter Experiment. (A) Experiment designed to resolve different components of complex biomolecular condensates; MT and Diffusion Filters can be switched on or off by turning on/off saturation or strong gradient. (B) Four different condensate constituents with overlapping signals can be selected using the appropriate combination of filters

To test the filtering capability of this experiment on multiple-component condensates, we mixed two intrinsically disordered domains of heterogeneous nuclear ribonucleoprotein C1 (hnRNPC1), namely, IDR1 and IDR2 (Note S3 and Figure S3A). These constructs readily phase separate, reaching the highest turbidity at a molar ratio of 2:1 (Figure S3B,C). Droplets were stabilized by preparation in 0.5% agarose. Two protein components (^15^N-labeled IDR1 and ^13^C-labeled IDR2) present in each phase yield four distinct protein states that can now be individually studied by NMR within the same sample. Using our multiplexing filter, we were able to resolve the signals from each component, even though their spectra fully overlapped (Figure 2A,B). Reassuringly, the sum of the signals stemming from aliphatic protons from dilute and condensed phases for both IDR1 and IDR2 almost perfectly match the spectrum acquired with only isotope filter/edit turned on. Subtle chemical shift differences between the two phases are noticeable in the aliphatic region of IDR1, especially at 0.8 and 1.1 ppm, demonstrating the high selectivity of the experiment. As an additional control, we acquired experiments in the presence of 200 mM NaCl that completely abrogated phase separation of IDR1 and IDR2, demonstrating only minor saturation of the dilute phase signal (10–20%, Figure S4A,B) akin to our simulations (Figure 1B). In yet another sample prepared using a different IDR1/IDR2 ratio, we detected vastly altered relative partitioning between the two phases (Figure S4C) with only a minor signal in the condensed phase. In conclusion, this illustrates the wide applicability of this methodology across very different condensed phase populations (Figures 1C,D, 2, and S4).

**Figure 2:**
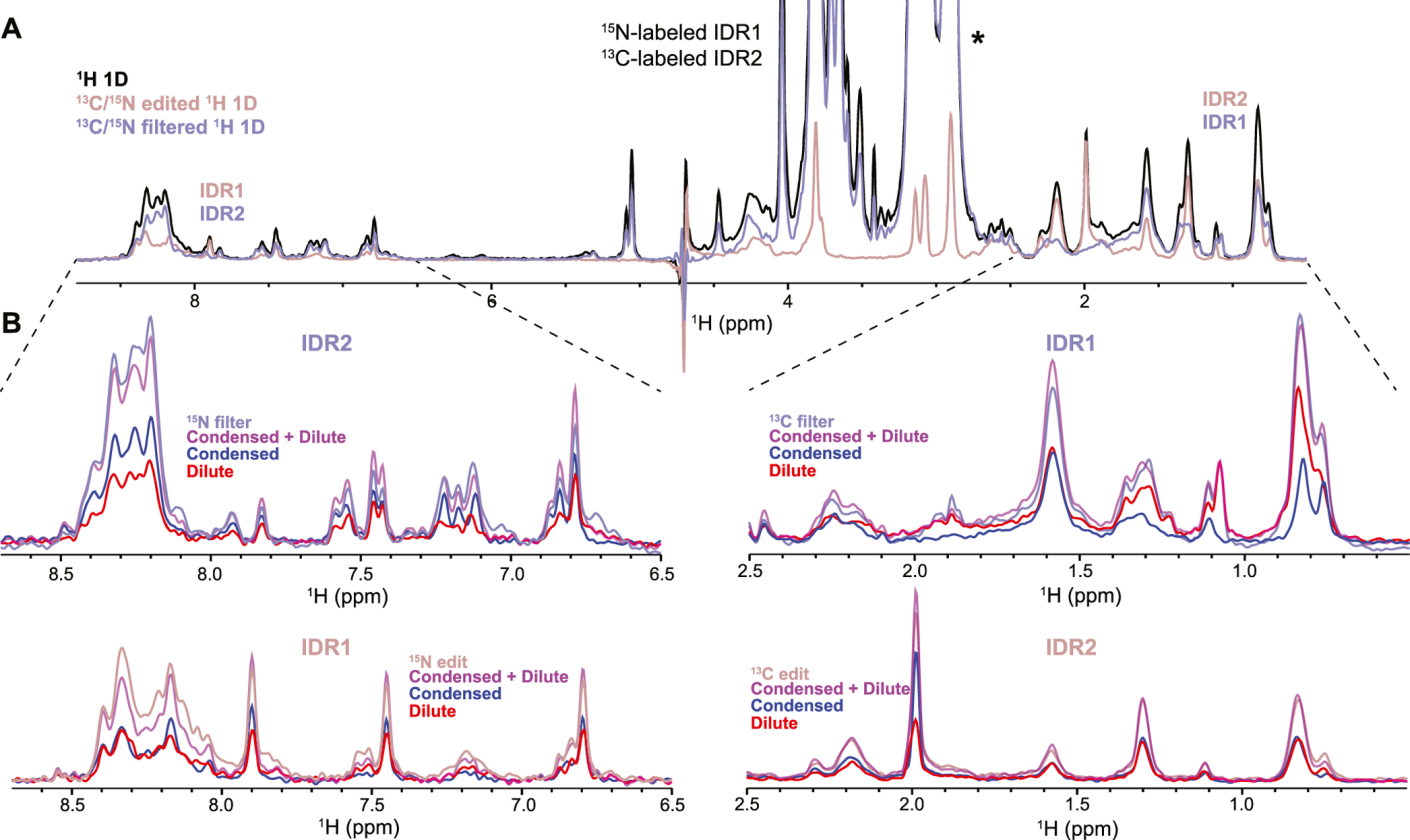
(A) Using an isotope-filter/edit block one can select different proteins according to their isotope enrichment. (B) Combined with isotope-filtering/editing, diffusion, and MT filters can further resolve overlapping signals stemming from condensed and dilute phases. For amide detection, aliphatic proton peaks are saturated and vice versa. The efficiency of selecting the full signal from both phases is highly efficient for aliphatic signals. For amide detection, the presence of chemical exchange can affect discrimination of both phases, obtained with diffusion and MT filter, leading to an underestimation of the respective populations. Signals labeled with * are stemming from HEPES buffer.

While the signals in the aliphatic region perfectly sum up to the total signal, this is not the case in the amide region due to the presence of chemical exchange and more extensive broadening in the condensed phase. The backbone amides are typically broadened by the slower global tumbling in the condensed phase, which, in addition to chemical exchange with water, can result in relaxation losses during the diffusion filter block. Furthermore, partial water saturation during the MT filter can also be transferred to exchanging amides and lead to further underestimation of the dilute phase. Therefore, special considerations need to be taken when studying labile protons. While, in theory, sufficiently fast exchange of biomolecules between condensed and dilute phases may similarly be detrimental for selective detection of their spectra, for IDPs, these exchange phenomena typically occur at a time scale too slow to considerably affect our filtering experiments.^41^ A more elaborate discussion on the effect of chemical exchange on our experiments is outlined in Note S4.

Naturally, the multiplexing filter can be combined with other multidimensional NMR experiments to analyze the structure and dynamics of the different protein pools. Unlike the diffusion filter, which introduces an additional relaxation pathway and is highly susceptible to relaxation and water exchange,^42^ the MT filter can be applied without relaxation losses (Figure S5A,B). This raised the idea to use the MT contrast to indirectly obtain the ^15^N–^1^H amide correlations stemming from the condensed phase by interleaving the irradiation between on- and off-resonances and performing difference spectroscopy. While the *T*_2_ contrast might be in principle similarly utilized for condensed phase selection, we observed similar line widths for condensed and dilute phase amide proton signals in this particular system. Based on our simulations, this renders *T*_2_ filter experiments inefficient (Note S5 and Figure S6). We therefore integrated our MT-based filter within an ^15^N–^1^H HSQC experiment to acquire the dilute phase and, via MT difference spectroscopy, the condensed phase spectrum. We recorded spectra at 28.2 T field strength (1.2 GHz ^1^H frequency) to exploit enhanced spectral resolution and a larger frequency separation (in Hz) of the cw saturation offset from the water resonance, thus minimizing spurious saturation (Notes S1 and S4). Figure 3A,B shows ^15^N–^1^H correlations of IDR1 from the two phases at 298 and 288 K, respectively, resolved from highly overlapping spectra. Zoomed insets demonstrate excellent selectivity of the MT filter, especially at 288 K where the cross-relaxation in the condensed phase becomes even more efficient due to slower dynamics. In both cases, a much better SNR was obtained compared to the diffusion-filtered experiments (Note S6 and Figure S7). Given the results in Figure 2B, this approach is similarly applicable for observing the ^13^C–^1^H correlations in the condensed phase.

**Figure 3:**
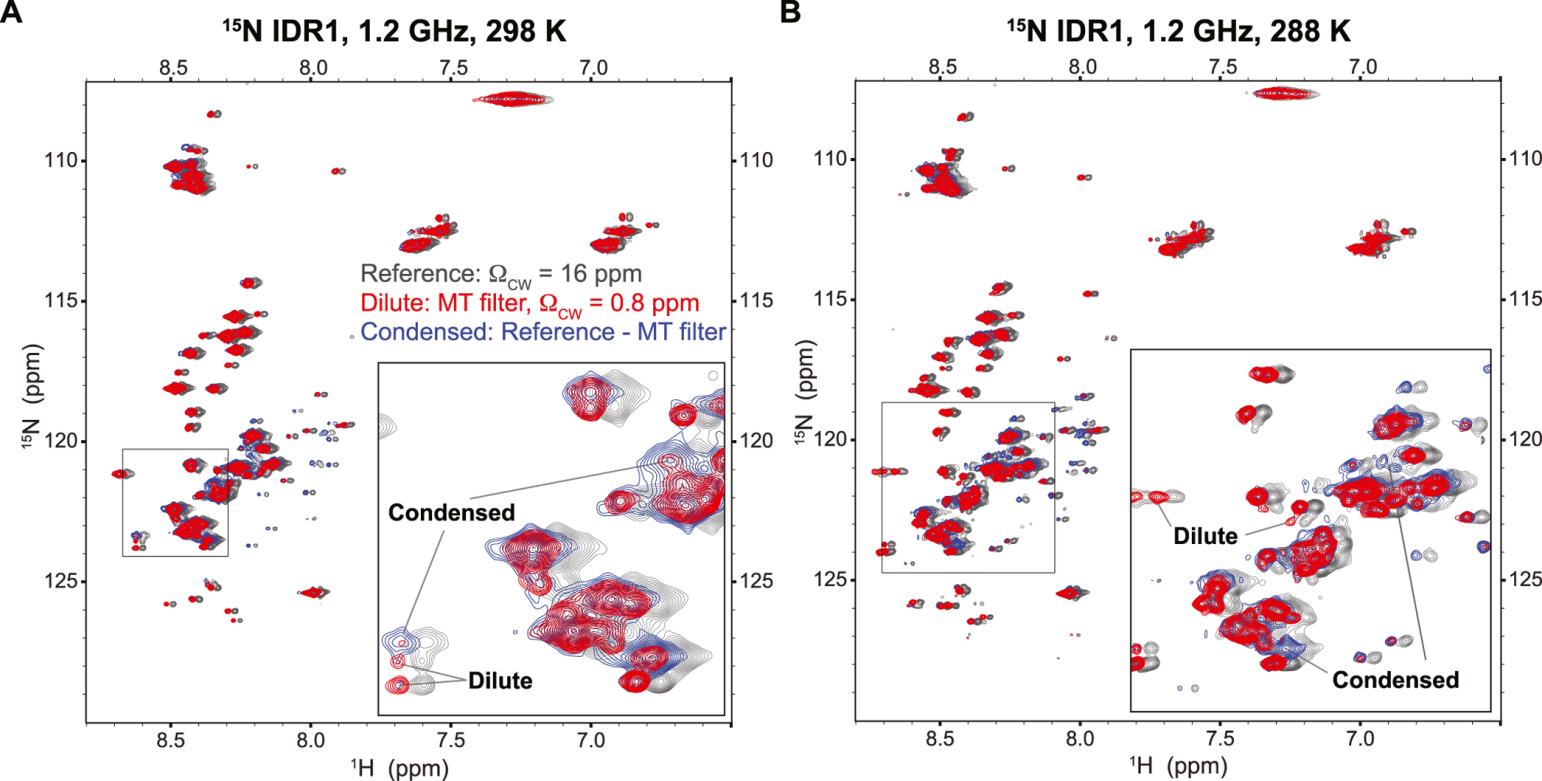
^15^N–^1^H correlations of IDR1 stemming from dilute and condensed phase acquired at (A) 298 K and (B) 288 K. Condensed phase spectra were obtained as a difference between MT-filtered HSQC with saturation at 0.8 ppm and a reference spectrum where saturation is applied off resonance at 16 ppm. This offset was chosen to be downfield from amides at the same distance as the on-resonance saturation of aliphatic protons to correct for spurious saturation. Although heavily overlapped, zoomed insets highlight differences between dilute and condensed phase peaks. Reference spectra were arbitrarily shifted by −0.025 ppm in ^1^H dimension for clarity.

## Conclusion

Our results demonstrate that the multiplexing filter can be used for “multicolor” analysis of protein–protein condensates resolving up to four components of a biphasic phase-separated system. We envision that the multiplexing capabilities can be extended with yet another component, which is a doubly ^15^N- and ^13^C-labeled molecule. In this case, for example, the aliphatic protons can be selected through multiple ^1^H–^15^N–^13^C–^1^H bond transfers, which might ultimately allow the analysis of up to six different molecular pools. Although not yet investigated, the same principle is applicable to RNA molecules, as these are very often an integral part of biomolecular condensates. The experiment is easy to set up, nondestructive, and allows in vitro analysis of biological condensates at near-physiological conditions. Discussed extensions of the current method and application in more complex environments are currently being investigated.

## Supplementary Material



## Notes

Additional experimental details, theoretical description, optimizations, and limitations (PDF)

## Abbreviations

NMR: nuclear magnetic resonance
IDR: intrinsically disordered region
RBD: RNA-binding domain
LLPS: liquid–liquid phase separation
MT: magnetization transfer
HSQC: heteronuclear single quantum coherence
