# Supplementary material for: Multiplexed Analysis of Multicomponent Biomolecular Condensates without Any Tag
Source: J Am Chem Soc. 2025 Dec 8;147(51):47237–43. doi: 10.1021/jacs.5c14476 (PMC12752461; doi:10.1021/jacs.5c14476)
Supplement: Supplementary file 1 [file ja5c14476_si_001.pdf]

Supporting Information for

Multiplexed analysis of multicomponent biomolecular condensates without any tag

Gyula Pálfy,[1,‡] Johannes Schmoll,[1,‡] Maria E. Pérez,[1] Fred F. Damberger,[1] Yaning Han,[1] Leonidas Emmanouilidis,[1] Frédéric H-.T. Allain[1,*] & Mihajlo Novakovic[1,*]

[1]Department of Biology, Institute of Biochemistry, ETH Zurich, Zurich, Switzerland

[‡]Gyula Pálfy and Johannes Schmoll contributed equally.

This PDF file includes:

Supporting Information Notes

Figures S1 to S7

Supporting Information References

Note 1: T_2 filter efficiency and magnetization transfer simulations

Although there is a notion that protein signals significantly broaden in the highly dense phase in biomolecular condensates (as is the case with amide backbone resonances), the differences are less pronounced for side chain ^{1}H signals, especially for intrinsically disordered regions of proteins. Preserving the fast local dynamics, side chains from IDRs usually remain highly dynamic even in the condensed phase. This leads to very subtle differences in both T_1[1] and T_2 relaxation parameters[2] between the two phases, rendering them nearly indistinguishable based on relaxation parameters. Filtering out fast-relaxing species by applying a spin echo is historically widely used, however the downside of this filter is that it monotonically suppresses also the desired slowly relaxing signals. When relaxation constants are not significantly different as in the case of IDRs in condensates, this can lead to a suppression of both species. This is simulated in Figure S1A. Based on the amide linewidths that we observed for IDR1-IDR2 biphasic sample (Figure S6), we chose two different combinations of T_2 relaxation constants, 50 and 33 ms (solid-colored lines) as well as a more favorable case for the T_2-filter of 80 and 20 ms (dashed-colored lines). Typically, one would choose 20, 40 or 70 ms echo delay (vertical dashed lines in Figure S1A). Clearly, in every case, a T_2 filter leads to significant reduction of signals from both phases and one can only achieve the near quantitative suppression of the condensed phase at 70 ms echo time. However, this would lead to almost 60% reduction of the dilute phase, making the T_2-filter far from ideal for applications with biomolecular condensates.

On the other hand, Figure S1B illustrates the experimental performance of the MT filter applied to FUS NTD construct. By combining both MT and diffusion filter, one can assess the residual

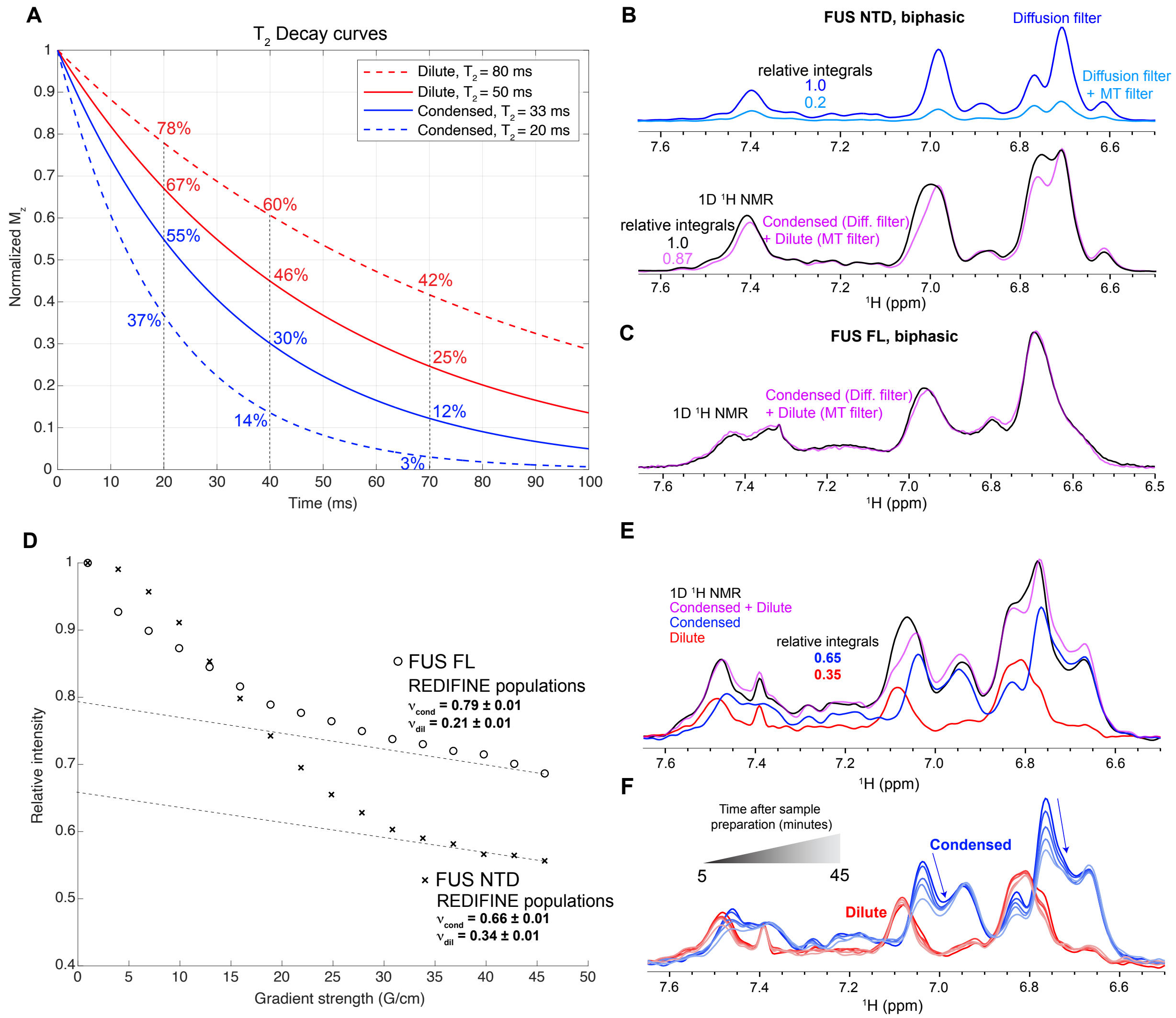

Figure S1. (A) Simulation of the filtering-efficiency of a T_2-filter experiment at various echo times and relaxation constants. Relaxation constants are realistically chosen according to the previously measured data and literature values. (B) Reduction of condensed phase signal upon application of an MT filter. As simulations in Figure 1B illustrated, MT can effectively suppress 80% of the condensed phase signal before reaching a steady state while reducing the dilute phase by ca. 15%. 5 s irradiation with a nutation field of 1000 Hz at 0.8 ppm (2720 Hz away from water at 700 MHz spectrometer) was used for the MT filter (C) The sum of the two phases is indistinguishable from the total ^{1}H 1D signal (acquired using the same stimulated echo, but with diffusion gradients and MT filter off) for the FUS FL protein condensates. Here we used 3s irradiation with a nutation field of 1000 Hz, centered at -0.3 ppm (4500 Hz away from water at 900 MHz spectrometer). (D) Signal decay in DOSY experiment (short 75 ms diffusion delay and 10 ms diffusion gradients) can be used to estimate the population of protein in condensed phase. (E) Diffusion and MT filter performance in the absence of agarose. (F) Evolution of dilute phase and condensed phase signal over time. Note that only the condensed phase signal is gradually reduced due to sedimentation while the dilute phase signal remains constant.

condensed phase that cannot be suppressed by the MT filter even under the steady state condition. This demonstrates that the MT filter effectively suppressed ≥80% of the condensed phase,

consistent with the simulation shown in Figure 1B performed using τ_C of 20 ns and r_{HH} of 2.3 Å. Furthermore, by comparing the total 1D ^{1}H signal and the sum of the selected signal from the two phases obtained with the MT and the diffusion filter respectively (Figure S1B), we can see that the dilute phase is slightly underestimated by ca. 13% due to the much less efficient but still present cross-relaxation within the protein in the dilute phase. This is consistent with the prediction in Figure 1B using τ_C of 4 ns and r_{HH} of 3 Å. To compensate for relaxation losses during the diffusion filter and MT filter blocks, total 1D ^{1}H signal is assessed by acquiring the spectrum using the same length echo time, but with gradients and cw saturation switched off. Surprisingly, a similar comparison yields almost indistinguishable spectra for the FUS FL protein (Figure S1C). The better reproduction of total signal upon summing the sub-spectra in the case of FUS FL protein means that the MT filter affected FUS FL protein in the dilute phase to a lesser extent compared to FUS NTD. Although many different parameters can play a role, potential contributions to this slight difference might come from their different oligomerization propensities in the dilute phase as well as the application of the same cw field strength at different magnetic fields (900 MHz for FUS FL vs. 700 MHz for FUS NTD). This achieves similar saturation efficiency (as it is long enough to reach steady state) but is more selective at higher fields due to the larger distance in Hz from water and other protein resonances. This effectively reduces spurious self-saturation of water and protein signal, increasing the overall efficiency of the MT filter.

Exact quantification of relative protein partitioning can be performed using our previously introduced REDIFINE method.[1] We therefore assessed whether the relative signal intensities shown in Figure 1C,D represent the relative populations of the protein in the condensed and dilute phase obtained by REDIFINE analysis.[2] While the data for FUS NTD match with the populations determined from signal intensities in the diffusion-filtered and MT filter spectra, the condensed phase of FUS FL is underestimated, while the dilute phase is correspondingly overestimated in Figure 1D. In general, the condensed phase signal also decays during the diffusion filter, and the MT filter cannot completely suppress the condensed phase signal (usually 80-90% is suppressed). It is therefore important to note that the condensed phase is usually underestimated while the dilute phase is slightly overestimated by simple comparison of the 1D filtered spectra. Therefore, we conclude that our multiplexing filter can qualitatively separate the two phases and is applicable only semi-quantitatively for determination of relative protein partitioning.

To simulate the differences in MT efficiency between the two phases we utilized a Bloch-McConnell-Solomon model[3] involving a two-spin system. This involved two protons H_A and H_B representing nearby aliphatic and aromatic/amide protons, connected via a generic cross-relaxation process, where one of them has been saturated using cw irradiation. The resulting equations can be written as:

$$\frac{dM_y^A}{dt} = \omega_{1A} M_z^A - R_2^A M_y^A$$

$$\frac{dM_z^A}{dt} = -\omega_{1A} M_y^A - (R_1^A + \sigma) M_z^A + \sigma M_z^B + R_1^A M_{eq}^A$$

$$\frac{dM_z^B}{dt} = -(R_1^B + \sigma) M_z^B + \sigma M_z^A + R_1^B M_{eq}^B$$

where M_y^A, M_z^A and M_z^B are the magnetization components of the proton spins along the specified axis of the Bloch sphere and M_{eq}^A and M_{eq}^B correspond to the equilibrium magnetizations of these reservoirs (for simplicity normalized to unity). Longitudinal and transverse relaxation rates were calculated as the inverse of the corresponding relaxation times. The strength of applied saturation field on proton H_A was denoted as ω_{1A}.

The dipole-dipole cross-relaxation rate describing the magnetization transfer between two spins is given by

$$\sigma = \frac{1}{10} b^2 (\mathcal{J}(0) - 6\mathcal{J}(2\omega^0))$$

where $\mathcal{J}(\omega) = \frac{\tau_c}{1+\omega^2\tau_c^2}$ is the spectral density function, and $b = -\frac{\mu_0}{4\pi}\frac{\hbar\gamma^2}{r_{HH}^3}$ is the dipole-dipole coupling constant. The strength of the saturation field applied along the x-axis is denoted as w_{1A}. For the simulation shown in Figure 1, we used the following parameters: $\omega_{1A} = 500$ Hz, $T_1^A = 0.3$ s, $T_1^B = 0.6$ s, $T_2^A = 0.05$ s, $r_{HH} = 3$ Å or 2.3 Å. The change in interproton distance upon condensation was chosen based on ~20% compaction in the condensed phase reported by Schuler and coworkers.[4] $\tau_c = 4$ ns is chosen for dilute phase and $\tau_c = 10 - 50$ ns to account for slower tumbling in the condensed phase.[2,5] Simulations are performed for $B_0 = 16.3$ T ($\omega_{\mathrm{H}} = 700$ MHz).

Note 2: Multiplexing filter pulse sequence – practical guide

The pulse sequence for the multiplexing filter is shown in Figure S2. Briefly, it incorporates an MT filter (saturation to suppress condensed phase), diffusion filter (strong gradient to suppress dilute phase), and isotope filter/edit block to select isotope unlabeled or labeled components. All filters can be switched on and off depending on what is desired to be observed (Scheme 1 – main text). Solvent is suppressed with excitation sculpting during the last block. Importantly, the length of the entire multiplexing block was kept constant when acquiring different molecular pools, ensuring a constant total attenuation due to relaxation, assuming T_2 ^{1}H of all states are similar. This preserves the ratio of integrals of the different components in their individual spectra.

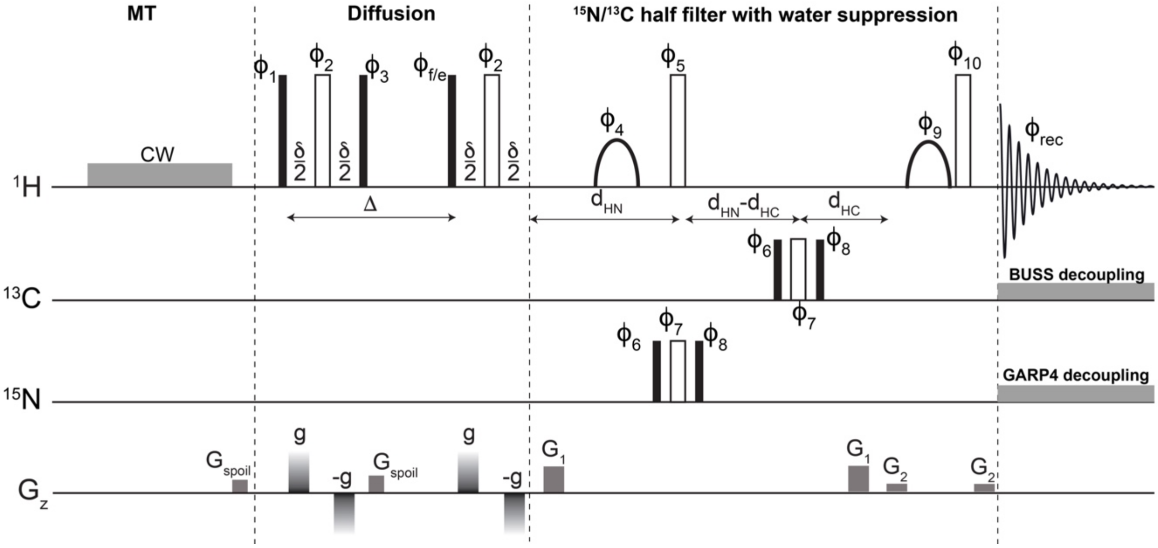

Figure S2. Pulse sequence for the multiplexing filter. Filled and outlined rectangles represent $\pi/2$ and π pulses respectively (except the outlined pulses on the X channels which are defined below). Δ is the diffusion delay while 2δ is the total duration of 4 gradients during the stimulated spin-echo. Delays are defined as $d_{HN}=1/2J_{NH}$ and $d_{HC}=1/2J_{CH}$. Outlined rectangles on ^{13}C and ^{15}N channels are defined as 2.66x the $\pi/2$ pulse on the same channel as a part of a composite π pulse. The two shaped pulses are selective rectangular-shape 2 ms π pulses on the water resonance which in combination with the G_1 and G_2 gradients achieve excitation sculpting for solvent suppression. Phases are defined as: ϕ_1=x, ϕ_2=x, ϕ_3=x, and depending on whether filter (f) or edit (e) flag is selected ϕ_f=8(x), 8(y), 8(-x), 8(-y) or ϕ_e=4(x), 4(-x), 4(y), 4(-y), 4(-x), 4(x), 4(-y), 4(y), ϕ_4=x, y, ϕ_5=-x, -y, ϕ_6=x, ϕ_7=y, ϕ_8=4(x), 4(-x), ϕ_9=x, x y, y, ϕ_{10}=-x, -x, -y, -y, ϕ_{rec}=2(x, -x, -x, x), 2(-y, y, y, -y), 2(-x. x, x, -x), 2(y, -y, -y, y). G_{spoil} gradients serve to suppress residual transverse magnetization (1 ms each of 40% and -17.13% of the maximum amplitude, respectively), g is the variable gradient used in the stimulated echo of the diffusion filter (2% when off and 95% when on), while G_1 and G_2 are for the excitation sculpting scheme (1 ms at 31% and 11%). The ^{15}N/^{13}C half-filter with integrated excitation sculpting is based on a pulse sequence written by Alvar Gossert (ETH Zurich).

MT filter

If aliphatic signals upfield from water are desired, then the cw irradiation can be applied on amides downfield from water. As a rule of thumb, the irradiation can be centered between 8 and 9 ppm and the nutation field applied with B_1 field strength of 700-1000 Hz, for 0.5-1 s. To ensure the steady state, we applied the irradiation for 3-5 s in Figure 1, however this is usually not necessary. In Figure 2, we used 1 s irradiation. This achieves effective suppression of the condensed phase in the aliphatic region without pronounced self-saturation of the aliphatic peaks. Similarly, if aromatic and/or amide protons are desired, cw irradiation should be placed at 0 or 1 ppm which can effectively suppress the aliphatic protons. The MT filter selectivity benefits from higher fields due to reduced spurious water saturation (larger absolute frequency difference from water).

Diffusion filter

Parameters of the diffusion filter vary greatly with the protein length. As a rule of thumb, to filter out dilute phase signals, one can apply 50-75 ms diffusion delay and 10-12 ms gradient duration at >40 G/cm depending on the probe capabilities. To limit the effect of chemical exchange (dilute-condensed protein and proton exchange with bulk water), the diffusion delay should be as short as possible. When the T_2 relaxation of the protein of interest is fast, the standard 10-12 ms gradient duration dictating the minimum echo time can lead to significant loss of signal – in these cases a shorter gradient duration should be chosen at the expense of longer diffusion time (relying on longer longitudinal relaxation). In other words, there is an interplay between these two parameters that needs to be optimized depending on the system. Optimization of these parameters can be performed using a standard stimulated echo DOSY experiment.

$^{15}N/^{13}C$ filter/edit block

We used a time-shared double half filter[6] that serves as $^{15}N/^{13}C$ filter/edit block resulting in the cleanest selection according to the isotope-label while optimizing sensitivity. The delays are tuned according to protein one bond amide J_{NH} and aliphatic J_{CH} couplings. Composite pulses are used on the X channels. The half filter is combined with an excitation sculpting scheme for efficient water suppression. BUSS and GARP4 decoupling are utilized for ^{13}C and ^{15}N channels respectively.

Note 3: hnRNPC1 IDRs readily phase separate

hnRNPC1 is a crucial RNA-binding protein involved in various aspects of RNA metabolism. Besides the RRM and leucine zipper domains, this protein contains two disordered regions, IDR1 and IDR2 (Figure S3A). When IDR1 and IDR2 are mixed, they readily phase separate with the highest turbidity at the ratio of 2:1. As a model system, we prepared a biphasic sample of these condensates stabilized by 0.5% agarose gel using ^{15}N-labeled IDR1 and ^{13}C-labeled IDR2 (Figure S3B,C), both of which remain observable in ^{1}H NMR upon phase separation and undergo slow chemical exchange between phases.

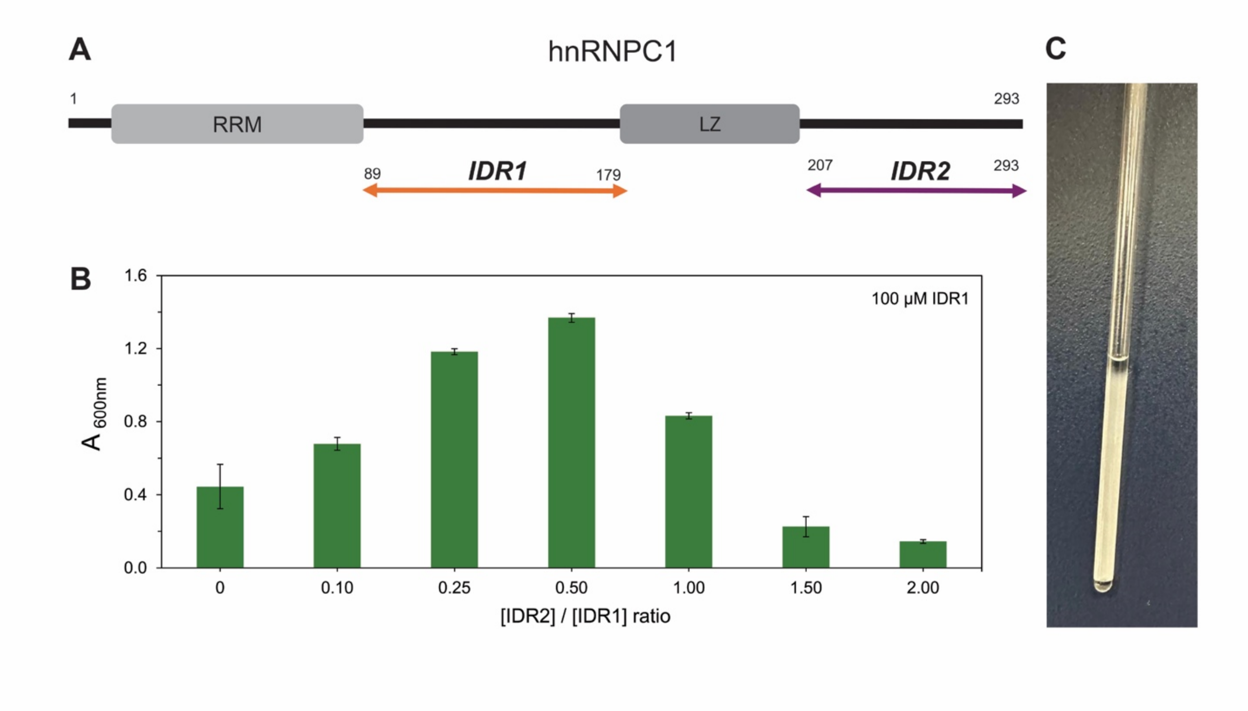

Figure S3. (A) Domain organization of the hnRNPC1 protein, illustrating the IDR1 and IDR2 domains used in this study. (RRM: RNA recognition motif, LZ: leucine-zipper domain, IDR1 and IDR2: disordered regions) (B) Turbidity assay of different IDR2/IDR1 molar ratio in the range of 0.0-2.0 using 100 µM IDR1 in 50 mM NaCl, 50 mM HEPES, pH 6.8. They undergo LLPS when mixed, yielding the highest turbidity at an IDR1/IDR2 ratio of 2:1. (C) A biphasic NMR sample (400 µM ^{15}N-IDR1, 200 µM ^{13}C-IDR2 in 50 mM NaCl, 50 mM HEPES, pH 6.0) prepared in 0.5% agarose is shown, in which pH was lowered to decrease the water exchange to obtain sharper signals in the spectra.

In addition to the sample with highest turbidity prepared at an IDR1/IDR2 ratio of 2:1 (Figure 2, main text), as controls we prepared the sample using the same stoichiometry but in presence of 200 mM NaCl that fully abrogated the condensate formation as well as another sample at the ratio of 1:1 that resulted in lower turbidity. Both samples were prepared in 0.5% agarose. Serving as a

dilute phase control, the high-salt, non-LLPS sample demonstrated that the protein in dilute phase is only marginally affected by MT filter (Figure S4A,B), as predicted by our simulations in Figure 1B. The slightly larger reduction of IDR1 signal upon irradiation can be attributed to the presence of its homo-oligomeric states that most likely contribute to the exchange-relayed spin-diffusion. Consistent with the absence of condensates in this sample, there is no signal in the diffusion-filtered condensed phase spectrum.

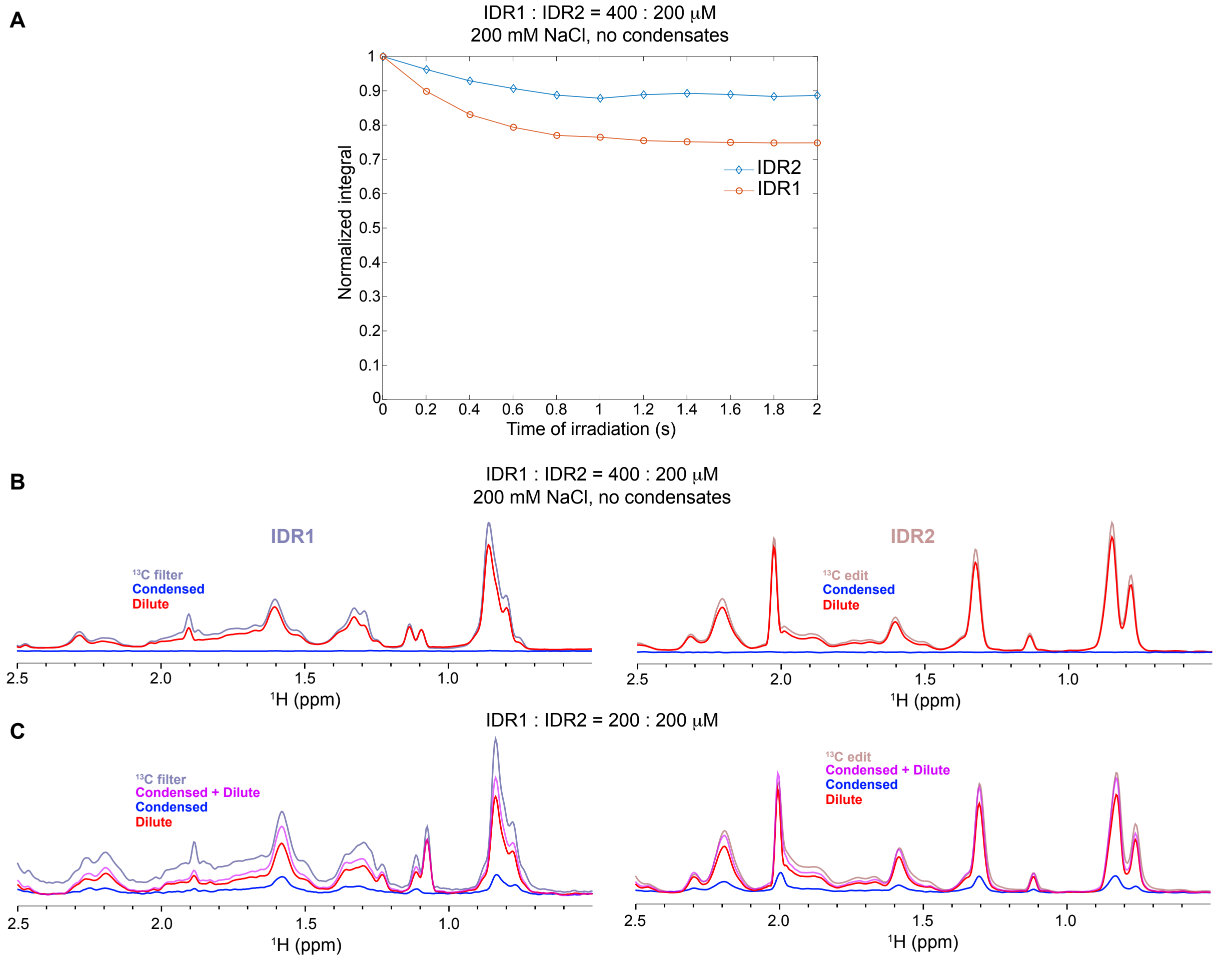

Figure S4. (A) Experimental data acquired on IDR1-IDR2 sample prepared in 0.5% agarose in presence of 200 mM NaCl that abrogates phase separation. (B) Spectra acquired using 0.5 s irradiation illustrate the slight reduction in signal. As there is no condensed phase in this sample, there is no signal in the diffusion-filtered spectrum. (C) sample prepared at IDR1 to IDR2 ratio of 1:1 containing notably reduced populations in the condensed phase as expected from the turbidity plot (Figure S3) and correspondingly much stronger signals in the dilute phase.

Furthermore, at an IDR1:IDR2 ratio of 1:1, we detected very low protein populations in the condensed phase (as anticipated from turbidity measurements in Figure S3), while the MT-filtered spectrum yielded correspondingly strong signals which compensated for this (Figure S4C).

Note 4: Chemical exchange can complicate the diffusion and MT filter

Chemical exchange, either between two phases or proton exchange with bulk water, can be detrimental for the efficiency of the MT and diffusion filter.[7,8] Sufficiently fast interphase exchange of biomolecules may compromise the distinction of condensed and dilute phase spectra via both MT- and diffusion-filtering, and ultimately yields exchange-averaged spectra. The timescale on which exchange may affect our MT-filter is defined by the irradiation time (which is in the order of a second). Exchange rates, derived from reported FRAP recovery half times measured for relevant condensates formed by IDRs (4 s for FUS, 2.5 s for DDX4, and 15 s for TDP43),[9] are smaller than 0.3 s^{-1} suggesting that interphase exchange will not cause significant effects on the MT filter. However, faster exchange rates could effectively relay saturated protein from the condensed to the dilute phase.

In contrast, exchange of labile protons with water typically occurs on a faster timescale than exchange of biomolecules between dilute and dense phase. This is why special care needs to be taken when targeting amides. The potential problem can be visualized when comparing a diffusion-filtered ^{15}N-^{1}H HSQC with the regular ^{15}N-^{1}H HSQC. One can immediately see that exchanging amide signals decay somewhat faster in a DOSY experiment,[7,8] however the real problems can be pinpointed in the diffusion-filtered ^{15}N-^{1}H HSQC (Figure S5A). Note that the diffusion-filtered HSQC suppresses also many peaks that can be associated with the condensed phase and preserves only the signals with slowest T_2 relaxation and exchange.

What is more, the presence of self-assembling species in the dilute phase which can exchange with the protein monomers in the dilute phase can lead to further underestimation of the dilute phase in the MT filter experiment. This is due to exchange-relayed spin-diffusion effects; however this is not the general phenomenon and only occurs in specific systems.

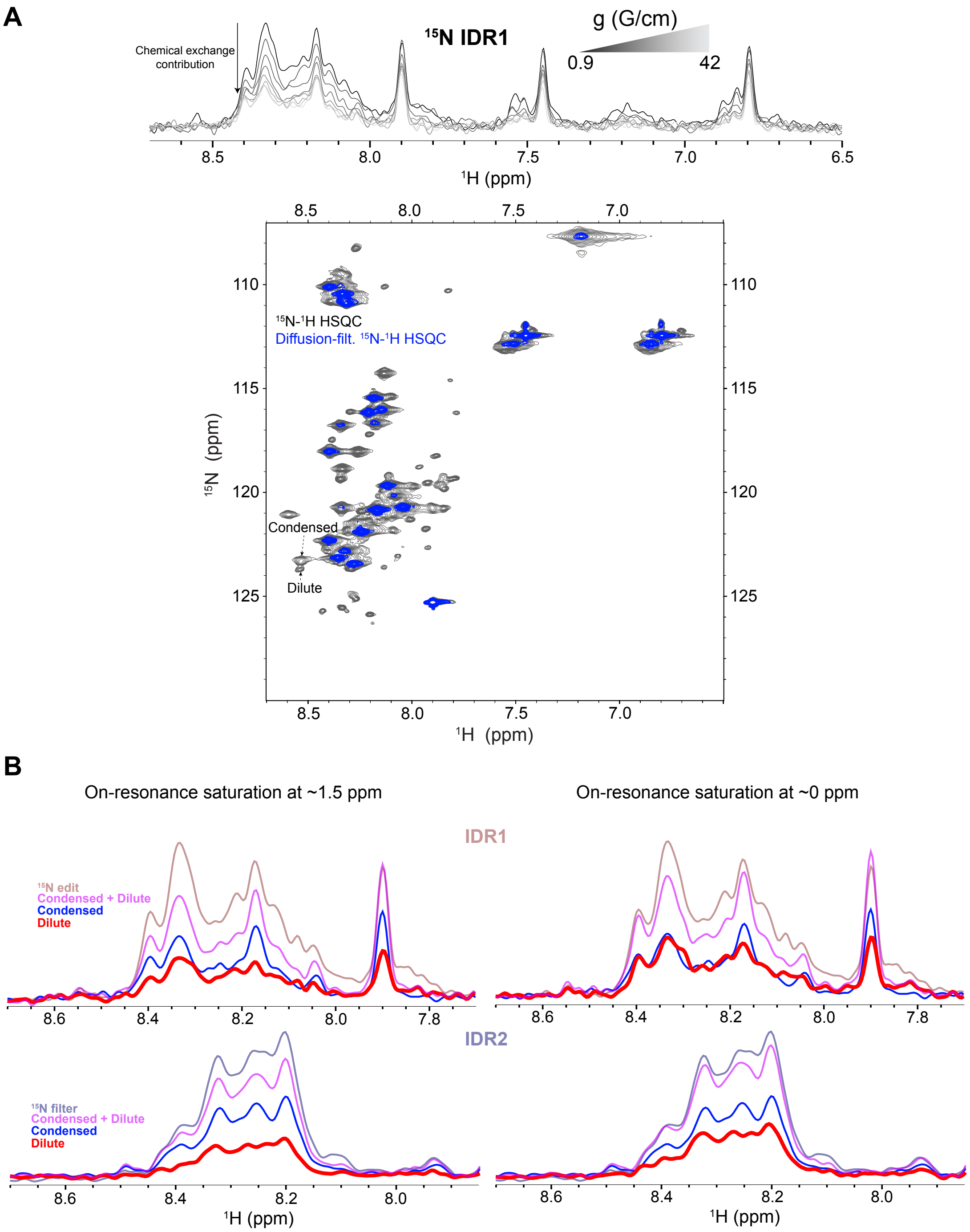

Figure S5. (A) ^{15}N-edited DOSY decay of IDR1 as a part of a condensate with IDR2 and the corresponding diffusion-filtered ^{15}N-^{1}H HSQC (50 ms diffusion delay, 12 ms gradient, 45 G/cm). Note that many signals are fully suppressed, even the ones that are known to stem from the protein in the condensed phase. (B) Shifting the irradiation from 1.5 to 0 ppm in the MT filter enhances the amides in the dilute phase due to the lesser extent of spurious water saturation.

Similarly, chemical exchange with water can affect the MT filter when selecting amides. Namely, spurious water saturation can be transferred to exchanging amide protons underestimating the signals stemming from dilute phase. It is therefore beneficial to irradiate at an offset as far as possible from water to reduce this effect (Figure S5B). This can be appreciated by the increase of the dilute phase signal and the fact that the sum of dilute phase and condensed phase spectra agree with the total proton signal when irradiation is performed at 0 ppm instead of 1.5 ppm. This is why higher fields are especially beneficial for the MT filter. At higher magnetic fields, aliphatic protons resonate further from water, allowing one to irradiate far enough from water, while still effectively saturating the protein (1 ppm on 700 MHz is 2600 Hz from water, while 3300 Hz on 900 MHz and 4400 Hz on 1.2 GHz).

Note 5: T_2 filter applicability on amide protons of IDR1

As pointed out in the main text, a condensed phase spectrum indirectly extracted from the difference of an MT-filtered and a reference spectrum may provide improved sensitivity and fewer signals suppressed due to spurious water saturation compared to diffusion filtered HSQC. If cw irradiation is executed first on-resonance with protein and then interleaved by a reference acquisition with a saturation pulse of the same B_1 intensity applied off resonance, one can acquire the condensed phase signal by calculating the difference spectrum of the on and off resonance saturation experiment. If amide protons have significantly different linewidths in the dilute and condensed phase, a T_2 contrast could in principle be exploited in a similar manner. In a ^{15}N-^{1}H HSQC spectrum of the ^{15}N-labeled IDR1 in biphasic mixture with IDR2 we could measure 17-25 Hz for ^{1}H signals in the dilute-phase and 30-50 Hz for signals in the condensed phase. For the purpose of simulations, we identified two resolved peaks, stemming from the same residue from the two different phases (Figure S6A). From the extracted 1D slices containing these resonances (Figure S6A inset) we could calculate that the condensed phase peak is approximately 32 Hz compared to 22 Hz in the dilute phase. Based on these linewidths, we used the corresponding T_2 relaxation constants to simulate the performance of the T_2 filter (Figure S6B). Note again that even with backbone amide protons there is no ideal spin-echo delay to effectively select the pure dilute phase component.

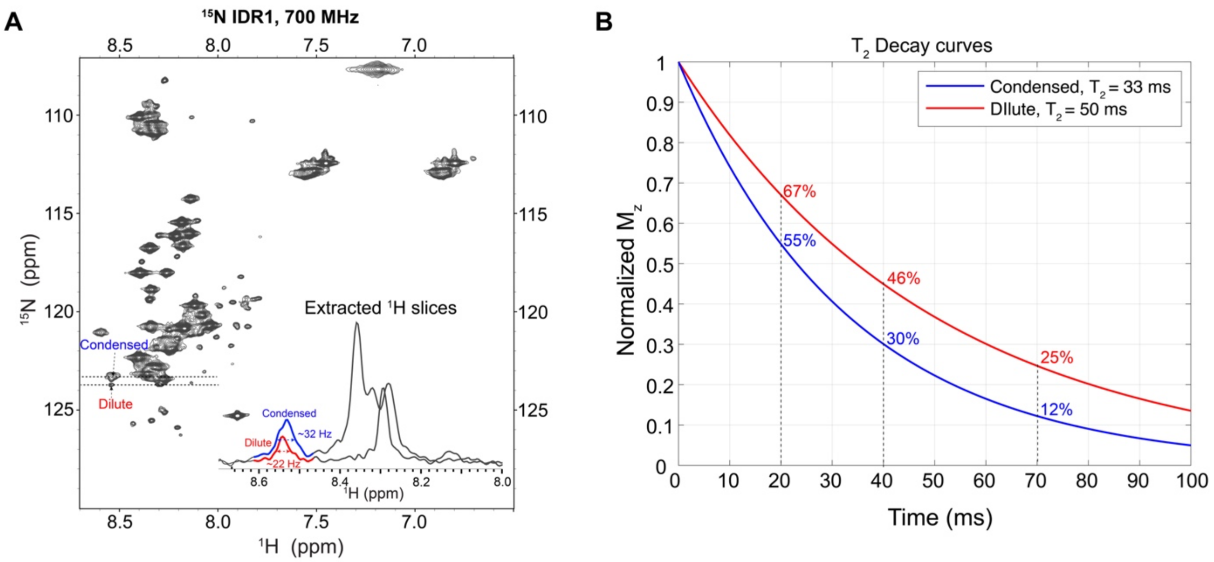

Figure S6. (A) ^{15}N-^{1}H HSQC of ^{15}N-labeled IDR1 in the biphasic sample with IDR2. The dashed lines point to spectroscopically resolved resonances stemming from the dilute and condensed phase signal of the same residue. 1D slices extracted from the specified ^{15}N chemical shifts (123.3 and 123.7 ppm), illustrating slightly different linewidths of amides in the two phases (~22 Hz vs. ~32 Hz), are shown in the inset. (B) Given the amide linewidths estimated in (A), we can simulate the T_2 decay showing that T_2 filter is not ideal choice to select the dilute phase of this particular sample.

Note 6: The quality of the 2D amide condensed phase spectra

Besides chemical exchange, fast transverse relaxation during the stimulated echo period can further limit diffusion filter performance. This is especially pronounced in diffusion filtered heteronuclear correlation experiments where magnetization is subjected to additional coherence transfers. Figure S7A,B shows the comparison of diffusion-filtered ^{15}N-^{1}H HSQC spectra compared to the ones obtained using MT filter and utilizing difference spectroscopy at 298 K and 288 K. While the latter yielded the spectra of high quality at both temperatures, the diffusion-filtered spectra had very low SNR with the detrimental effect of fast T_2 relaxation especially pronounced at 288 K. In contrast, slower dynamics at 288 K rendered cross-relaxation even more efficient, which further improves the performance of the MT filter, illustrating that one could further enhance the efficiency of the multiplexing filter by changing the temperature.

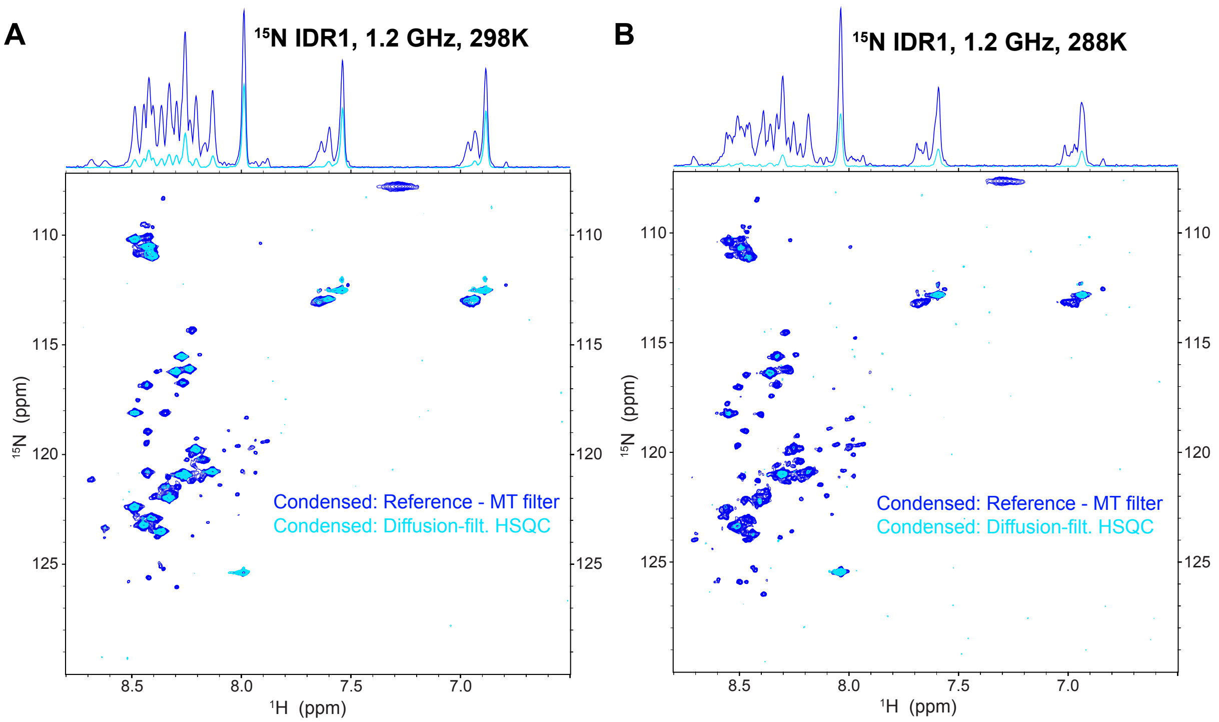

Figure S7. Comparison between the condensed phase ^{15}N-^{1}H HSQC spectrum of IDR1, obtained by MT filtered difference spectroscopy and the diffusion filter, at (A) 298 K and (B) 288K. Although requiring 2-times more scans for acquisition, the condensed phase extracted from difference spectroscopy has far superior performance compared to the diffusion filter. This is especially pronounced at 288 K where faster T_2 relaxation due to slower tumbling proves detrimental for diffusion filter (due to the presence of relatively long echo). 1D spectra on the top represent ^{1}H positive projections.

References

(1) Novakovic, M.; Han, Y.; Kathe, N. C.; Ni, Y.; Emmanouilidis, L.; Allain, F. H.-T. LLPS REDIFINE Allows the Biophysical Characterization of Multicomponent Condensates without Tags or Labels. *Nat Commun* **2025**, *16* (1), 4628. https://doi.org/10.1038/s41467-025-59759-2.

(2) Brady, J. P.; Farber, P. J.; Sekhar, A.; Lin, Y. H.; Huang, R.; Bah, A.; Nott, T. J.; Chan, H. S.; Baldwin, A. J.; Forman-Kay, J. D.; Kay, L. E. Structural and Hydrodynamic Properties of an Intrinsically Disordered Region of a Germ Cell-Specific Protein on Phase Separation. *Proc Natl Acad Sci U S A* **2017**, *114* (39), E8194–E8203. https://doi.org/10.1073/pnas.1706197114.

(3) Novakovic, M.; Kupče, Ē.; Oxenfarth, A.; Battistel, M. D.; Freedberg, D. I.; Schwalbe, H.; Frydman, L. Sensitivity Enhancement of Homonuclear Multidimensional NMR Correlations for Labile Sites in Proteins, Polysaccharides, and Nucleic Acids. *Nat Commun* **2020**, *11* (1), 5317. https://doi.org/10.1038/s41467-020-19108-x.

(4) Galvanetto, N.; Ivanović, M. T.; Chowdhury, A.; Sottini, A.; Nüesch, M. F.; Nettels, D.; Best, R. B.; Schuler, B. Extreme Dynamics in a Biomolecular Condensate. *Nature* **2023**, *619* (7971), 876–883. https://doi.org/10.1038/s41586-023-06329-5.

(5) Najbauer, E. E.; Ng, S. C.; Griesinger, C.; Görlich, D.; Andreas, L. B. Atomic Resolution Dynamics of Cohesive Interactions in Phase-Separated Nup98 FG Domains. *Nat Commun* **2022**, *13* (1), 1494. https://doi.org/10.1038/s41467-022-28821-8.

(6) Burgering, M.; Boelens, R.; Kaptein, R. Observation of Intersubunit NOEs in a Dimeric P22 Mnt Repressor Mutant by a Time-Shared [15N, 13C] Double Half-Filter Technique. *J Biomol NMR* **1993**, *3* (6), 709–714. https://doi.org/10.1007/BF00198373.

(7) Liu, M.; Toms, H. C.; Hawkes, G. E.; Nicholson, J. K.; Lindon, J. C. Determination of the Relative NH Proton Lifetimes of the Peptide Analogue Viomycin in Aqueous Solution by NMR-Based Diffusion Measurement. *J Biomol NMR* **1999**, *13*, 25–30.

(8) Thureau, P.; Ancian, B.; Viel, S.; Thévand, A. Determining Chemical Exchange Rates of the Uracil Labile Protons by NMR Diffusion Experiments. *Chemical Communications* **2006**, No. 2, 200–202. https://doi.org/10.1039/b513580j.

(9) McSwiggen, D. T.; Mir, M.; Darzacq, X.; Tjian, R. Evaluating Phase Separation in Live Cells: Diagnosis, Caveats, and Functional Consequences. *Genes Dev* **2019**, *33* (23–24), 1619–1634. https://doi.org/10.1101/gad.331520.119.
